# EPAC1 Pharmacological Inhibition with AM-001 Prevents SARS-CoV-2 and Influenza A Virus Replication in Cells

**DOI:** 10.3390/v15020319

**Published:** 2023-01-23

**Authors:** Charlotte Foret-Lucas, Thomas Figueroa, Alexandre Bertin, Pierre Bessière, Alexandre Lucas, Dorian Bergonnier, Marine Wasniewski, Alexandre Servat, Arnaud Tessier, Frank Lezoualc’h, Romain Volmer

**Affiliations:** 1 Ecole Nationale Vétérinaire de Toulouse, Université de Toulouse, ENVT, INRAE, IHAP, UMR 1225, 31300 Toulouse, France; 2 Institute of Metabolic and Cardiovascular Diseases, INSERM, Université de Toulouse, UMR 1297-I2MC, 31432 Toulouse, France; 3 Nancy Laboratory for Rabies and Wildlife, ANSES, Lyssavirus Unit, 54220 Malzéville, France; 4 Nantes Université, CNRS, CEISAM, UMR 6230, 44000 Nantes, France

**Keywords:** SARS-CoV-2, influenza A, host-targeted antiviral, EPAC1 inhibitor, cylic AMP

## Abstract

The exceptional impact of the COVID-19 pandemic has stimulated an intense search for antiviral molecules. Host-targeted antiviral molecules have the potential of presenting broad-spectrum antiviral activity and are also considered as less likely to select for resistant viruses. In this study, we investigated the antiviral activity exerted by AM-001, a specific pharmacological inhibitor of EPAC1, a host exchange protein directly activated by cyclic AMP (cAMP). The cAMP-sensitive protein, EPAC1 regulates various physiological and pathological processes but its role in SARS-CoV-2 and influenza A virus infection has not yet been studied. Here, we provide evidence that the EPAC1 specific inhibitor AM-001 exerts potent antiviral activity against SARS-CoV-2 in the human lung Calu-3 cell line and the African green monkey Vero cell line. We observed a concentration-dependent inhibition of SARS-CoV-2 infectious viral particles and viral RNA release in the supernatants of AM-001 treated cells that was not associated with a significant impact on cellular viability. Furthermore, we identified AM-001 as an inhibitor of influenza A virus in Calu-3 cells. Altogether these results identify EPAC1 inhibition as a promising therapeutic target against viral infections.

## 1. Introduction

COVID-19, the disease caused by the severe acute respiratory syndrome coronavirus 2 (SARS-CoV-2), killed millions of people worldwide and the death toll is increasing as the virus is still circulating [1]. Tremendous efforts have been made to develop efficient therapeutics against COVID-19, and several treatments have been shown to reduce the transmission of COVID-19 [2]. However, there is still an urgent need for more effective antiviral drugs to treat SARS-CoV-2 infection and further decrease the spread of the disease.

The cyclic nucleotide adenosine 3′,5′ cyclic monophosphate (cAMP) is a universal second messenger that relays external signals from membrane receptors to regulate a multitude of physiological and pathological processes [3]. Cyclic AMP is produced from ATP by membrane or soluble adenylyl cyclase (AC), while phosphodiesterases catalyze its hydrolytic degradation into the inactive 5′-AMP. In multicellular eukaryotic organisms, protein kinase A (PKA) and the exchange proteins directly activated by cAMP (EPACs) are predominantly mediated by intracellular effectors of cAMP. The two isoforms of EPAC, EPAC1 and EPAC2 are guanine nucleotide exchange factors that directly activate the small G proteins, Rap1 and Rap2 upon binding of cAMP [4]. EPAC1 is a ubiquitous protein, highly expressed in the heart and kidney whereas EPAC2 has a more restricted pattern of expression in the central nervous system and endocrine tissues [5]. Compelling evidence indicates that EPACs play key regulatory roles in controlling various biological functions including cell division, growth, and apoptosis. Consistently, analyses of EPAC1 and EPAC2 knockout mouse models revealed the importance of these cAMP-sensitive proteins in the development of various diseases including heart failure, cancer, diabetes, and inflammation [5,6].

Of particular importance, the development of specific pharmacological probes targeting EPACs without affecting PKA activity has also greatly facilitated the characterization of the biological role of EPAC in diverse cellular systems [5,7]. Among the few selective EPAC1 inhibitory compounds identified, CE3F4 (*R*-enantiomer), a tetrahydroquinoline analogue and AM-001, a thieno [2,3-b]pyridine derivative were characterized as an EPAC1-specific noncompetitive antagonist and EPAC1-uncompetitive antagonist, respectively [8,9,10]. These small molecules are efficient at high concentrations of cAMP. In addition, besides their use as pharmacological tools to study EPAC1 signaling routes, in vitro and in vivo experiments revealed the therapeutic properties of CE3F4 and AM-001 in various experimental models of cardiac disease [10,11]. However, the potential role of selective EPAC1 antagonists in the context of viral infections has not yet been investigated.

There are very few studies on the involvement of cAMP-EPAC signaling axis in viral infections. An early paper showed that pretreatment with ESI-09, a pan-EPAC small molecule antagonist that inhibited both EPAC1 and EPAC2 isoforms, decreased replication of Middle East Respiratory Syndrome coronavirus (MERS-CoV) and SARS-CoV in vitro [12]. Further studies demonstrated that EPAC1 genetic inhibition inhibits Ebola virus infection in both ex vivo vasculature and endothelial cells independently of the classic cAMP-PKA signaling pathway [13]. Interestingly, it was recently suggested that EPAC2 may influence different respiratory viral infections from EPAC1 such as those induced by human metapneumovirus, adenovirus infections, and respiratory syncytial virus [14,15].

The role of EPACs in SARS-CoV-2 or influenza A virus infection has not yet been explored. Therefore, we investigated whether selective pharmacological inhibition of EPAC1 isoform with the AM-001 compound could regulate SARS-CoV-2 replication using Vero E6 and Calu-3 epithelial airway cells.

## 2. Materials and Methods

### 2.1. Synthesis of AM-001

The chemical structure of AM-001 is based on a thieno [2,3-b]pyridine scaffold composed of three structural units containing thiophenyl, phenyl, and fluorophenylamide moieties. The synthesis of this class of compound was described previously [16,17] and the AM-001 synthesis was carried out from 3-phenyl-1-(2-thienyl)prop-2-en-1-one. The first step of synthesis involved a cyclization reaction in the presence of cyanoacetamide under basic conditions. These reaction conditions produced, as expected, 4 phenyl-3-cyano-6-(2-thienyl)pyridine-2(1H)-thione with a moderate yield of 40%. Having in hand the pyridine-2(1H)-thione as an intermediate derivative, the subsequent intramolecular Thorpe-Ziegler cyclization was carried out in a one-pot reaction in the presence of N-4-fluorophenyl-2-chloroacetamide. After final purification, the AM-001 derivative was cleanly isolated with a yield of 85%.

### 2.2. Cell Culture and Viral Infections

Human lung epithelial cells Calu-3 (ATCC HTB-55) were cultured in Dulbecco’s Modified Eagle Medium: Nutrient Mixture F-12 (DMEM:F12) containing 20% fetal calf serum (FCS) and GlutaMAX™ Supplement 1X (Gibco). Vero E6 cells (ATCC CRL-1586) were cultured in Dulbecco modified Eagle’s minimal essential medium (DMEM) containing 10% FCS. The pre-variants of concern strain of SARS-CoV-2 (hCoV-19/France/OCC-IHAP-VIR12/2020) used in this study was isolated from a nasal swab, kindly provided by the Toulouse hospital (CHU Toulouse Purpan, France), following two passages in Vero E6 cells. For the SARS-CoV-2 infection and evaluation of the antiviral properties of AM-001, Calu-3 cells were plated 48 h before treatment and infection in 12-well plates at a density of 10^6^ cells per well. The H1N1 influenza A/PR/8/34 (NIBSC vaccine strain), kindly provided by Ron A. Fouchier [18] (Erasmus Medical Center, Rotterdam, Netherlands) was grown and titrated in Madin-Darby Canine Kidney (MDCK) cells (ATCC CCL-34). Cells were infected at a multiplicity of infection of 0.001 with SARS-CoV-2 in DMEM:F12 containing 2% FCS or with influenza A virus Opti-MEM medium (Gibco) containing 0.5µg/ml L-(tosylamido-2-phenyl)ethyl chloromethyl ketone (TPCK) treated trypsin.

### 2.3. Treatments with AM-001 and Remdesivir

Unless otherwise stated, cells were pretreated for two hours with AM-001 at different concentrations or with 6 µM Remdesivir (Tocris, UK). Following this pretreatment, the medium was removed and the cells were infected in medium containing viruses and AM-001 or Remdesivir. After one hour of virus inoculation, cells were washed twice with PBS and the cell culture medium was replaced with infection medium containing AM-001 or Remdesivir at 6 µM.

For the time-of-addition assay, treatments with 20 µM AM-001 or 6 µM Remdesivir were initiated at −2, 0, or 4 h relative to SARS-CoV-2 infection. After one hour of virus inoculation, cells were washed twice with PBS and the cell culture medium was replaced with infection medium containing AM-001 or Remdesivir at 6 µM.

### 2.4. Quantification of Viral Load in Cell Supernatants

For SARS-CoV-2, viral RNA was extracted 24 h post-infection from the supernatants of infected cells and copy numbers of the viral gene E were determined by TaqMan OneStep RT-qPCR with E_Sarbeco primers and probe [19] and following instructions of the QIAGEN QuantiNova Probe RT-PCR Kit. At 24 h post-infection, infectious particles were also titrated from the supernatants of infected cells by the tissue culture infectious dose 50% (TCID50) method on Vero E6 cells. For influenza A virus, infectious virus was titrated from the supernatants of infected cells 24 h post-infection by the TCID50 method on MDCK cells.

### 2.5. Cellular Viability Assay

Vero E6 and Calu-3 cells were plated 48 h before treatment in 96-wells plates at a density of 1.10^5^ cells per well. After 24 h of treatment with the different concentrations of AM-001, cellular viability was measured using the Cell Proliferation Kit I (MTT) (Roche Applied Science, Indianapolis, IN) according to the manufacturer’s protocol.

### 2.6. Virucidal Assay

SARS-CoV-2 viral stock at 4 × 10^6^ TCID50/ml was exposed to AM-001 at 0.2 µM, 2 µM, or 20 µM or to an equivalent concentration of DMSO (vehicle treated control NT) or to 0.1% Triton X-100 (positive control for virucidal activity) in cell culture medium for one hour at 37 °C. The mix was then diluted 10^4^-fold and this dilution added to Vero E6 cells for titration by the TCID50 method, as previously reported [20].

### 2.7. Bioluminescence Resonance Energy Transfer (BRET) Assay

BRET experiments were performed to monitor EPAC1 activation as previously described [8]. Briefly, VeroE6 cells were transfected with the EPAC1-BRET probe CAMYEL. One day after transfection, cells were lysed in a buffer containing HEPES (40 mM), KCL (140 mM), NaCl (10 mM), MgCl2 (1.5 mM), and Triton (0.5%) supplemented with protease and phosphatase inhibitors. After centrifugation at 16,000 *g* for 20 min, cell supernatants were collected and loaded into a 96-well plate. AM-001 (20 µM) and coelenterazine-h (2μM) were added 7 min before injection of cAMP (100 µM)

### 2.8. Immunoblot Assay

Cells were washed twice with PBS and scraped with 500 µL lysis buffer (HEPES 40 mM, KCL 140 Mm, NaCl 10 mM, MgCl2 1.5 mM and 0.5% Triton X-100) supplemented with protease and phosphatase inhibitors. The lysates were then centrifuged at 16,000 g for 20 min at 4 °C and denatured at 95 °C for 5 min. After denaturation, different protein concentrations of the lysates were loaded and separated in a 12% SDS-polyacrylamide gel. Proteins were transferred into a nitrocellulose membrane using the Trans-blot turbo transfer apparatus (20 V, 1 A) for 30 min. The membrane was blocked in TBS-Tween/3 % BSA for one h before the O/N incubation at 4 °C with the EPAC1 antibody (dilution 1:1000 in 3% BSA; Cell Signaling, Danvers, MA, USA). After washes with TBS-Tween 20, the membrane was incubated with an anti-mouse HRP coupled antibody 1 h30 at RT. The membrane was finally revealed by chemiluminescence using GE-Healthcare Amersham ECL Kit and read with BioradChemDocTM XRS. GAPDH (Cell Signaling).

### 2.9. Statistical Analyses

All the data were statistically analyzed using one-way analysis of variance (ANOVA) followed by Dunnett’s post hoc test (GraphPad Prism Software, MA, USA) with the *p* values indicated to the corresponding to the results of Dunnett’s post hoc test.

## 3. Results

### 3.1. Inhibition of SARS-CoV-2 by AM-001

We first verified that EPAC1 protein is expressed in Vero and Calu-3 cells by western-blot analysis (Figure S1A). Furthermore, we verified that AM-001 was indeed able to inhibit cAMP-induced EPAC1 activation by using a bioluminescence resonance energy transfer (BRET) assay in Vero cells (Figure S1B).

**Figure 1:**
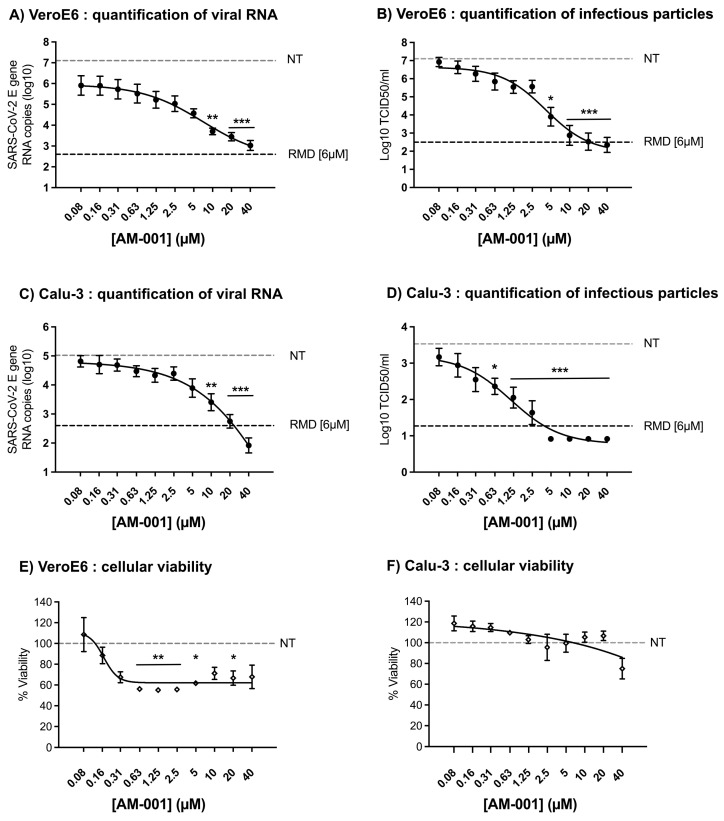
**Inhibition of SARS-CoV-2 replication in VeroE6 and Calu-3 cells treated with AM-001**. Cells pretreated for two hours with the indicated concentrations of AM-001 or with Remdesivir (RMD) at 6 µM or with an equivalent concentration of DMSO (non-treated control NT) were infected at a multiplicity of infection of 0.001 with SARS-CoV-2. After one hour of virus inoculation, the cell culture medium was removed and replaced with cell culture medium containing AM-001 at the indicated concentrations or Remdesivir at 6 µM. Quantification of viral RNA levels (**A**) and infectious particles (**B**) from VeroE6 cells 24 h post-infection. Quantification of viral RNA levels (**C**) and infectious particles (**D**) from Calu-3 cells 24 h post-infection. Quantification of cellular viability from VeroE6 (**E**) and Calu-3 (**F**) cells treated with the indicated concentrations of AM-001. Results are expressed as means ± SEM from at least three independent experiments. Statistical analysis: one-way ANOVA with Dunnett’s multiple comparisons test (* indicates *p* < 0.05; ** indicates *p* < 0.01 and *** indicates *p* < 0.001 compared to NT cells). The dotted gray line corresponds to the mean result from NT cells. The dotted black line corresponds to the mean result from RMD-treated cells.

Next, we evaluated the antiviral properties of AM-001 against SARS-CoV-2 by pre-treating Vero and Calu-3 cells before infecting them at a multiplicity of infection (MOI) of 10^−3^. As a positive control, we used remdesivir, which was shown to be a potent SARS-CoV-2 inhibitor in cell culture [21]. We verified that remdesivir caused a concentration-dependent inhibition of SARS-CoV-2 replication in Vero cells (Figure S2) and used it in all experiments at a concentration of 6 µM, corresponding to approximately ten times the half-maximal inhibitory concentration (IC_50_) [21]. Viral growth was measured 24 h post-infection by quantifying infectious titres and viral RNA in the supernatants. Remdesivir significantly inhibited viral growth when compared to control non-treated cells (NT). We observed a concentration-dependent reduction in viral RNA and infectious titres in the supernatants of Vero E6 treated with AM-001, with the AM-001 IC_50_ values of 388 nM and 355 nM against viral RNA production (Figure 1A) and infectious virus production (Figure 1B), respectively. In Vero cells treated with 20 µM AM-001, we detected a 10^4^-fold reduction in viral RNA levels and infectious titres compared to NT cells, reaching inhibition levels observed in cells treated with 6 µM remdesivir. Similar results were obtained in Calu-3 cells in which AM-001 IC_50_ against viral RNA production was 147 nM (Figure 1C) and the IC_50_ against infectious virus production was 167 nM (Figure 1D). Interestingly, AM-001 modestly reduced cellular viability in Vero cells (Figure 1E) and had no effect on the viability of Calu-3 cells (Figure 1F), indicating that inhibition of viral replication was not due to AM-001-induced cellular toxicity. The concentration of AM-001 causing a 50% reduction in cellular viability (CC_50_) could not be determined in our experiments (CC_50_ > 40 µM), suggesting that AM-001 has a selectivity index >100 in Vero and Calu-3 cells.

### 3.2. Time-of-Addition-Dependent Inhibition of SARS-CoV-2 by AM-001

**Figure 2:**
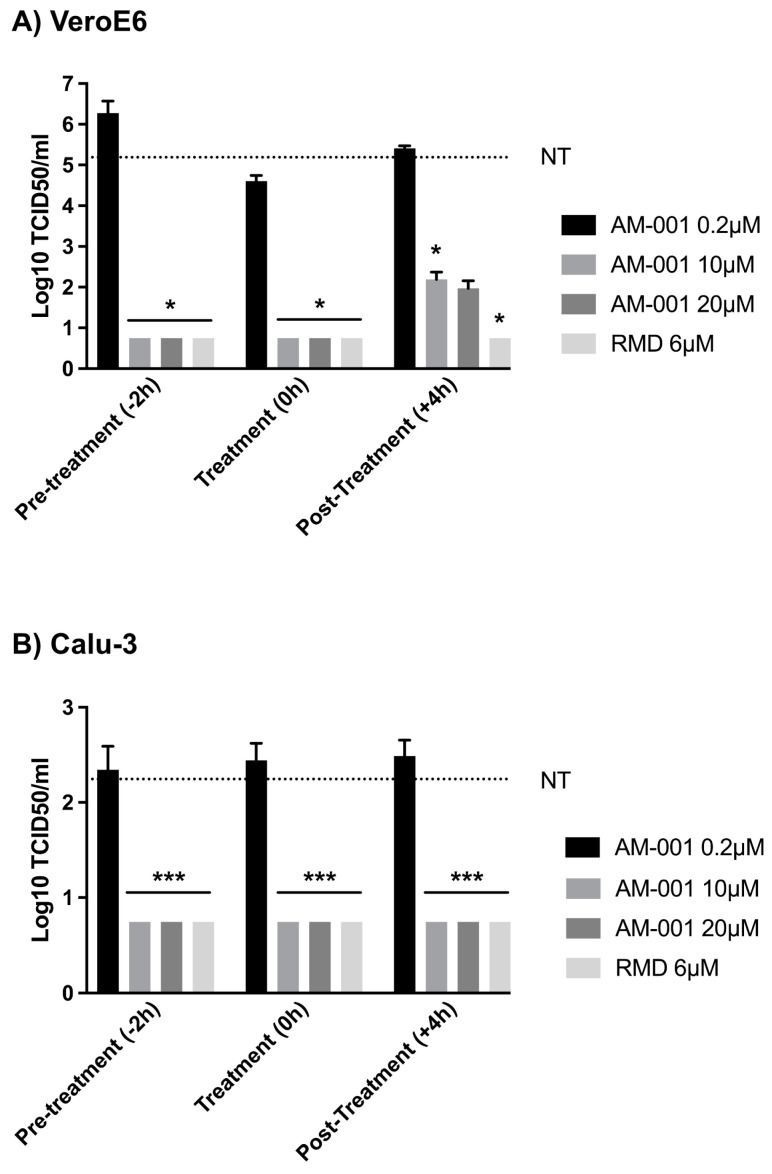
Evaluation of the antiviral properties of AM-001 against SARS-CoV-2 replication in VeroE6 and Calu-3 cells upon pre-treatment, treatment, or post-treatment. For the time-of-addition assay, treatments with 0.2 µM, 10 µM, or 20 µM AM-001 or 6 µM Remdesivir were initiated at −2, 0 or 4 h relative to SARS-CoV-2 infection. At 24 h post-infection, infectious particles in the supernatant of VeroE6 (**A**) and Calu-3 (**B**) cells were titrated using the TCID50 method. Results are expressed as means ± SEM from one representative experiment performed at least three times. Statistical analysis: one-way ANOVA with Dunnett’s multiple comparisons test (* indicates *p* < 0.05 and *** indicates *p* < 0.001 compared to NT cells). The dotted gray line corresponds to the mean result from NT cells.

To determine if the time of AM-001 treatment onset relative to the inoculation time modulated its antiviral properties, we performed a time-of-addition assay in which AM-001 was added to cells at −2, 0, or 4 h relative to SARS-CoV-2 infection. We found that 10 µM AM-001 inhibited infectious viral particles production as efficiently as 6 µM remdesivir when applied before infection or simultaneous to virus inoculation in Vero (Figure 2A) and Calu-3 cells (Figure 2B), and when AM-001 was added 4 h post-infection in Calu-3 cells (Figure 2B). However, in Vero cells treated 4 h post-infection, the antiviral properties of AM-001 did not match the level of inhibition obtained with 6 µM remdesivir, or when AM-001 was applied before infection or simultaneous to virus inoculation, suggesting that AM-001 may act on the early stages of viral infection (Figure 2A). The inhibition of viral growth was statistically significant in Vero cells treated 4 h post-infection with 10 µM AM-001, but not in Vero cells treated 4 h post-infection with 20 µM AM-001, when compared to non-treated cells.

### 3.3. Lack of Virucidal Activity of AM-001 against SARS-CoV-2

**Figure 3:**
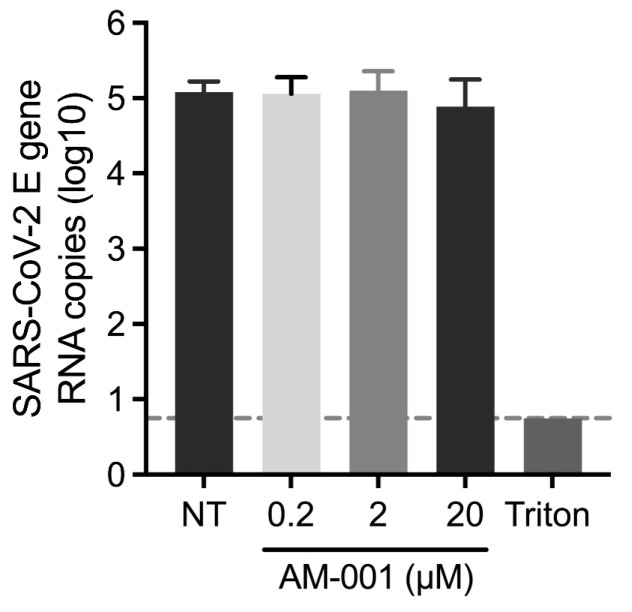
**Evaluation of the virucidal properties of AM-001 against SARS-CoV-2**. SARS-CoV-2 viral stocks were exposed to the indicated concentrations of AM-001, or to an equivalent concentration of DMSO (vehicle treated control NT), or to 0.1% Triton X-100 (positive control for virucidal activity) in cell culture medium for one hour at 37 °C. A 10^5^-fold dilution was subsequently used to titrate the infectivity of the treated viral stocks. Results are expressed as means ± SEM from one representative experiment performed at least three times. The dotted gray line corresponds to the limit of detection of the TCID50 assay. Statistical analysis performed with one-way ANOVA with Dunnett’s multiple comparisons test revealed no significant difference between vehicle treatment (NT) and AM-001 treatment.

To exclude the possibility that AM-001 inhibited SARS-CoV-2 in cell culture by inactivating viral particles in the cell culture medium, we evaluated the virucidal properties of AM-001. We exposed SARS-CoV-2 to concentrations of AM-001 ranging from 0.2 µM to 20 µM in cell culture medium for one hour at 37 °C. Next, we diluted the virus 10^5^-fold to perform titration with final concentrations of AM-001 far below those having shown any inhibitory effect. We did not detect any impact of pre-exposure of viral particles AM-001 demonstrating that the antiviral properties of AM-001 are not linked to virucidal properties (Figure 3).

### 3.4. Inhibition of Influenza A Virus by AM-001

**Figure 4:**
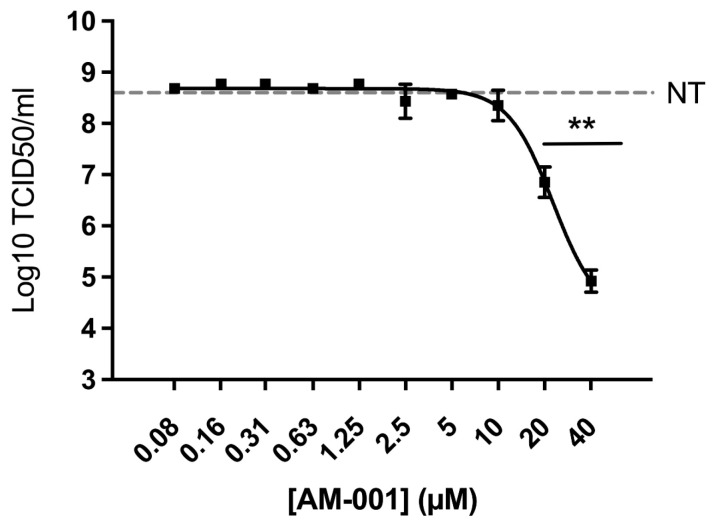
**Inhibition of influenza A virus replication in Calu-3 cells treated with AM-001**. Cells pretreated for two hours with the indicated concentrations of AM-001 or with an equivalent concentration of DMSO (non-treated control NT) were infected at a multiplicity of infection of 0.001 with influenza A virus. After one hour of virus inoculation, the cell culture medium was removed and replaced with cell culture medium containing AM-001 at the indicated concentrations. Infectious particles were measured 24 h post-infection. Results are expressed as means ± SEM from three independent experiments. Statistical analysis: one-way ANOVA with Dunnett’s multiple comparisons test (** indicates *p* < 0.01 compared to NT cells). The dotted gray line corresponds to the mean result from NT cells.

Finally, we evaluated the antiviral properties of AM-001 against influenza A virus. Viral growth was measured 24 h post-infection by quantifying infectious titres in the supernatants. We observed a concentration-dependent reduction in infectious titres in the supernatants of Calu-3 cells treated with AM-001, with an IC_50_ value of 1 µM (Figure 4).

## 4. Discussion

We provide evidence that the EPAC1-specific pharmacological inhibitor AM-001 is a potent inhibitor of SARS-CoV-2 and influenza A virus infection. The identification of AM-001 as a host targeted antiviral molecule against these pathogens, which are of major public health concern, should stimulate further research to evaluate the antiviral potency of AM-001 against other viral pathogens for which no therapeutics exist or that could develop resistance to viral-targeted therapeutics. Indeed, host-targeted antivirals are classically considered as less likely to select for resistant viral strains. In addition, they may offer broad-spectrum antiviral activity provided that the targeted host pathway is critical for the replication of various viruses belonging to different families.

Importantly, AM-001 has proven therapeutic properties in various experimental murine models of cardiac disease [10], indicating that the molecule was not degraded following in vivo intravenous or intraperitoneal administration and was not associated with any detectable toxic side effects in vivo. These observations provide strong impetus to evaluate the antiviral potency of AM-001 in animal models of viral infections.

A central question is to elucidate the AM-001 mechanism of action in the course of virus infection. Our finding that an EPAC1 pharmacological inhibitor prevents viral infection suggests that virus infection is probably linked to an increase in the intracellular production of cAMP since EPAC1 activity is activated by this cyclic nucleotide. This raises the question of the source of cAMP during viral infection. A possible explanation would be that virus entry could increase the pool of cAMP via the soluble adenyl cyclase (AC) which has been previously shown to regulate EPAC1 activation and endocytosis [22,23,24]. Consistently, it is suggested that soluble AC promotes endocytic pathway of viral entry by stimulating vacuolar ATPase-dependent acidification of endosomes and lysosomes [23]. Whether such a molecular event occurs in the context of SAS-CoV-2 or influenza A virus infection remains to be tested.

The time-of-addition studies revealed that AM-001 was less active in Vero cells when added postinfection than when cells were pretreated for two hours prior to infection or when treatment was initiated simultaneously to viral inoculation. These observations constitute a hint that AM-001 may act on the early stages of viral infection. In line with this hypothesis, inactivation of EPAC1 was shown to attenuate Ebola virus glycoprotein pseudotyped vesicular stomatitis virus entry into vascular endothelial cells [13]. Mechanistically, it is suggested that EPAC1 controls Ebola virus internalization via the regulation of macropinocytosis in a phosphatidylinositol-4,5-bisphosphate 3-kinase dependent-signaling pathway [13]. Of particular interest, EPAC1 and its downstream effectors, the small G proteins, Rap and Rho-GTPases are well-known regulators of the cytoskeletal components including actin and microtubule [25,26]. Because viral proteins modulate the structure and function of the actin cytoskeleton to initiate and spread infections [27], one could speculate that pharmacological inhibition of EPAC1 impairs viral entry into cells by modulating the dynamics of the cytoskeletal components. Whether such a molecular event or other steps of the virus host cell interaction are involved in the inhibition of viruses by AM-001 is still unknown. Thus, further studies are needed to elucidate the mechanism of action of AM-001 against SARS-CoV-3 and influenza A virus.

In conclusion, we have shown that EPAC1 pharmacological inhibition significantly impacts SARS-CoV-2 and influenza A viral replication, supporting that EPAC1 could be a promising therapeutic target against viral infection. Further studies are required to test the antiviral efficacy of EPAC1 pharmacological modulators in experimental animal models of SARS-CoV-2 and influenza A virus infection.

## Data Availability

Data available upon request to the corresponding authors.

## Supplementary Materials

The following supporting information can be downloaded at: https://www.mdpi.com/article/10.3390/v15020319/s1.
