# Supplementary material for: EPAC1 Pharmacological Inhibition with AM-001 Prevents SARS-CoV-2 and Influenza A Virus Replication in Cells
Source: Viruses. 2023 Jan 23;15(2):319. doi: 10.3390/v15020319 (PMC9965159; doi:10.3390/v15020319)
Supplement: Supplementary file 1 [file viruses-15-00319-s001.zip › viruses-2186375-supplementary.pdf]

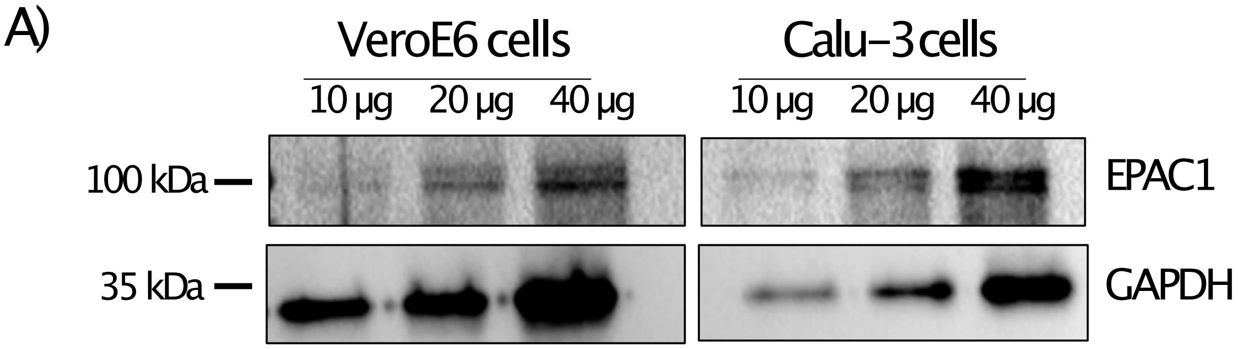


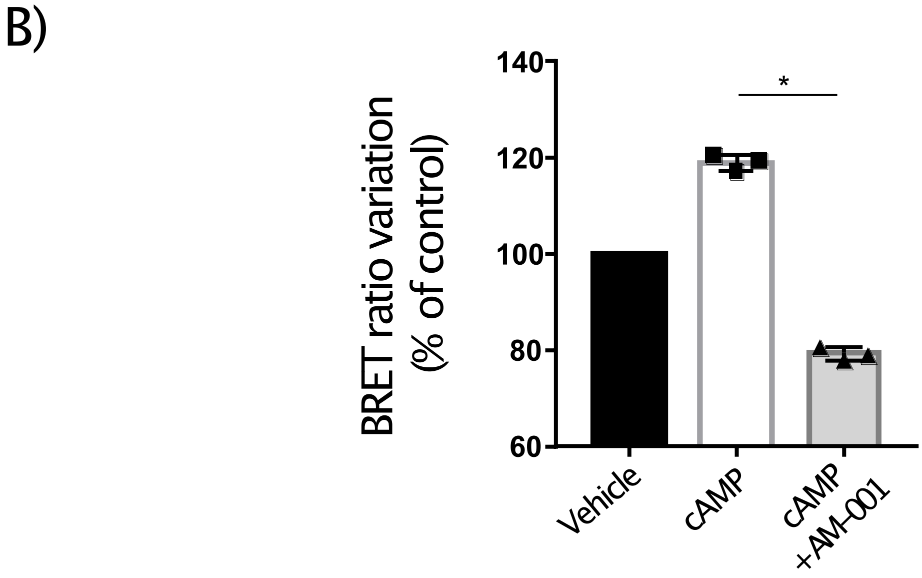


**Supplementary Figure S1. EPAC1 is expressed in VeroE6 and Calu-3 cells (A) and cAMP-dependent activation of EPAC1 is decreased with AM-001 as assessed by BRET assay (B).** (A) Immunoblot analysis showing the expression of EPAC1 at different concentrations of protein extracts. GAPDH, loading control. (B) EPAC1 activation was monitored by BRET assay in VeroE6 cells. AM-001 (20µM) were added to the cell extracts before addition of cAMP (100 µM), and BRET ratios were measured and plotted as percent variations in BRET ratios relative to non-stimulated control value. Values are mean ± SEM (triplicate). Statistical analysis: one-way ANOVA with Dunnett's multiple comparisons test (*< 0.05 vs vehicle or indicated value).

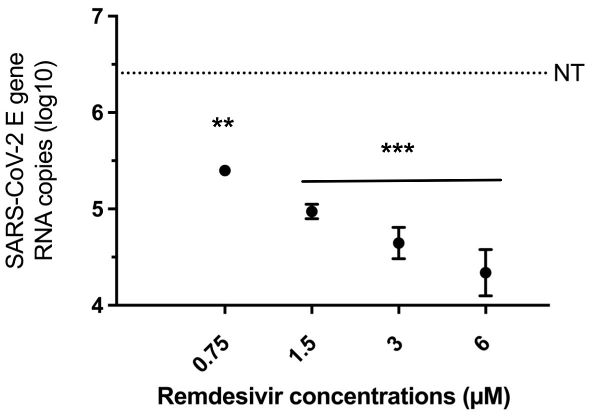


**Supplementary Figure S2. Inhibition of SARS-CoV-2 replication in VeroE6 treated with remdesivir**. Cells pretreated for two hours with the indicated concentrations of Remdesivir RMD at or with an equivalent concentration of DMSO (non-treated control NT) were infected at a multiplicity of infection of 0.001 with SARS-CoV-2. After one hour of virus inoculation, the cell culture medium was removed and replaced with cell culture medium containing remdesivir at the indicated concentrations. Quantification of viral RNA levels from VeroE6 cells was performed 24 hours post-infection. Results are expressed as means ± SEM from one representative experiment. Statistical analysis: one-way ANOVA with Dunnett's multiple comparisons test ** indicates P<0.01 and *** indicates P<0.001 compared to NT cells). The dotted gray line corresponds to the mean result from NT cells.
